# Predictors of permanent pacemaker requirement in aortic stenosis patients undergoing self-expanding valve transcatheter aortic valve replacement using the cusp overlap technique

**DOI:** 10.3389/fcvm.2025.1486375

**Published:** 2025-02-18

**Authors:** Yung-Tsai Lee, Tien-Ping Tsao, Kuo-Chen Lee, Huan-Chiu Lin, Chun-Ting Liu, Ming-Chon Hsiung, Wei-Hsian Yin, Jeng Wei

**Affiliations:** ^1^ Heart Center, Cheng Hsin General Hospital, Taipei, Taiwan; ^2^ Department of Exercise and Health Science, National Taipei University of Nursing and Healthy Science, Taipei, Taiwan; ^3^ Faculty of Medicine, National Defense Medical Center, Taipei, Taiwan; ^4^ Faculty of Medicine, School of Medicine, National Yang Ming Chiao Tung University, Taipei, Taiwan

**Keywords:** transcatheter aortic valve replacement, self-expanding valves, cusp overlap technique, conduction disturbances, permanent pacemaker implantation

## Abstract

**Introduction:**

Since TAVR was approved for lower-risk aortic stenosis (AS) patients, managing post-implantation conduction disturbances has become crucial, especially with self-expanding heart valves (SEV). This study aims to identify risk factors for conduction disturbances in such patients using a specific fluoroscopic cusp overlap (COL) technique.

**Methods:**

This retrospective study analyzed AS patients who underwent TAVR with SEV from 2019 to 2022, excluding those needing pacemakers or valve-in-valve procedures. Patients were grouped by conventional (CON) and COL techniques, with outcomes monitored using Valve Academic Research Consortium (VARC)-2 criteria.

**Results:**

In this cohort study of 114 patients, 17 were excluded due to pre-existing pacemakers. Forty-seven received SEVs using COL, and 50 with CON techniques. The COL group showed a significant reduction in new LBBB (27.7% vs. 46%, *p* = 0.006) and PPI rates (4.3% vs. 18%, *p* = 0.033) compared to the CON group. Deeper implantation depth below the non-coronary cusp (NCC) and left coronary cusp (LCC) was linked to an increased risk of conduction disturbances. Multivariate analysis identified smaller left ventricular outflow tract diameter, shorter membranous septum length, and greater pre-releasing implantation depth below the LCC as predictors of future PPI risk.

**Conclusion:**

The use of the COL technique significantly reduces the risk of newly developed conduction disturbances after SEV TAVR. Keeping SEV implantation depth within 1 mm of the membranous septum length and maintaining an implantation depth of <6 mm below the LCC before final release further minimizes the risk of PPI.

## Introduction

Since the approval of transcatheter aortic valve replacement (TAVR) for intermediate and low-risk aortic stenosis (AS) patients, lifetime management has become a significant concern, especially for younger and lower-risk individuals (1). One critical consideration in performing TAVR in these patients is the risk of conduction disturbances post-implantation, particularly with the use of self-expanding heart valves (SEVs) (2). Conduction disturbances following TAVR are associated with poor ventricular function recovery and increased hospitalizations years after the procedure (3, 4). These disturbances include new-onset LBBB and the need for permanent pacemaker implantation (PPI).

SEVs are associated with a higher risk of PPI compared to BEVs, primarily due to their longer stent frames and deeper implantation, which can disrupt the conduction system at the membranous septum. The use of right anterior oblique (RAO) fluoroscopic projection, known as the COL view, has been shown to reduce the incidence of PPI in patients undergoing SEV TAVR. Despite these advancements, a significant proportion of patients still require PPI after valve implantation. In the ATLAS registry, Kalogeras et al. reported a significantly lower PPI rate with the Evolut Pro™ compared to the Evolut R™ (5). However, data from the Society of Thoracic Surgeons (STS) registry (2014–2017) showed no significant difference in PPI rates across three generations of the CoreValve system (6). Recent findings on the Evolut FX suggest that PPI rates with this latest-generation valve are relatively low. These findings highlight ongoing uncertainty regarding whether advancements in device design alone are sufficient to reduce PPI rates (7). We propose that improvements in understanding the anatomy of the conduction system and optimizing implantation techniques, such as precise control of device depth, may also play a critical role in reducing PPI rates. This perspective underscores the need for further investigation and will be included in the introduction to provide a comprehensive background on PPI incidence following. This study aims to identify risk factors in AS patients undergoing SEV TAVR using the COL fluoroscopic projection in comparison to the conventional three-cusp (CON) view technique.

## Methods

### Patient population and data collection

This cohort study is a retrospective analysis conducted from January 1, 2019, to January 31, 2022, with institutional review board approval. The study enrolled AS patients who underwent TAVR using SEVs. From January 2019 to December 2020, the Medtronic CoreValve Evolut R™ (Medtronic, Minneapolis, MN) was the most commonly used device and was implanted using the conventional three-cusp (CON) view technique (the CON group). From January 2021 to December 2022, the Medtronic Evolut PRO™ (Medtronic, Minneapolis, MN) was the most commonly used device and was implanted using the COL technique (the COL group). Patients who required a pacemaker before TAVR and those undergoing aortic valve-in-valve procedures were excluded.

Pre-operative, peri-operative, and post-operative characteristics and outcomes were recorded according to Valve Academic Research Consortium (VARC)-2 criteria (8) up to 30 days after TAVR. Electrocardiograms (EKGs) were analyzed for PR interval, QRS interval, P axis, QRS axis, R axis, and QTc pre-operatively, immediately after TAVR, and 1-week post-TAVR implantation.

### Definitions

On an EKG, left bundle branch block (LBBB) is identified by a QRS complex duration of 120 ms (ms) or more, indicating delayed ventricular depolarization due to a blockage in the left bundle branch. Morphologically, LBBB typically shows a broad, notched, or slurred R wave in Lead V1 (rS or RSr' pattern) and a similarly broad, notched, or slurred S wave in Lead V6. The lateral leads (I, aVL, V5, V6) also exhibit broad, notched, or slurred R waves with a delayed intrinsicoid deflection (R peak time ≥60 ms). Right bundle branch block (RBBB) is characterized by a prolonged QRS complex duration, typically exceeding 120 ms, indicating altered conduction through the right bundle branch, affecting the sequence and timing of ventricular depolarization. In Lead V1, RBBB often presents with a broad, slurred S wave (rsR' pattern), while Lead V6 shows a wide, notched R wave. These electrocardiographic characteristics indicate RBBB's impact on ventricular activation.

Severe Leaflet or Annular Calcification was defined by specific criteria assessed via computed tomography (CT) imaging, including an Agatston score of more than 2,000 for the aortic valve leaflets and annulus. Membranous Septum Length was assessed by multi-slice CT, which provides detailed cross-sectional views of the heart. This method allows for precise evaluation of the anatomical dimensions of the membranous septum in relation to surrounding cardiac structures. In the context of Minimizing Depth According to the Membranous Septum (MIDAS) (9), the membranous septum length is determined by identifying the thinnest part of the interventricular septum on the axial plane image, typically aligned with the tricuspid annulus (7). This approach is crucial for assessing the optimal depth for interventions involving the membranous septum, such as valve replacements or procedures impacting the heart's conduction system.

### TAVR implantation

All patients in the cohort received general anesthesia for the TAVR procedure, performed under transesophageal echocardiography guidance. Transesophageal echocardiography was used to monitor for complications, assess valvular and paravalvular leakage, and evaluate post-procedure outcomes according to VARC-2 definitions.

#### Conventional three-cusp (CON) technique

The SEVs implanted starting from the virtual ring plane under the three-cusp view (10). The implanter adjusted the TAVR stent to maintain a position 3–6 mm below the lowest non-coronary cusp (NCC) inflow point by continuously adjusting the delivery catheter. Control pacing at a rate of 120 BPM was initiated when the second portion (node 2) CoreValve stent frame emerged from the capsule to the point of no return, after which the CoreValve was fully released. If the final valve position was not ideal, the valve was recaptured and repositioned to an appropriate depth. The final pre-releasing depths (PRD) of the NCC and left coronary cusp (LCC) were identified and recorded.

#### Cusp overlap (COL) technique

The valve was deployed at a higher position, from 1/2 to 1/3 of the diagnostic pigtail catheter at the nadir or NCC, using the fluoroscopic COL view (11). This view is achieved by adjusting the fluoroscopic angles so that the right coronary cusp (RCC) and LCC overlap, with the NCC centered between them, typically in a right anterior oblique (RAO) caudal position. After releasing the valve, it descends into the left ventricle and stops 1–6 mm below the annular virtual ring at the NCC side. At the point of no return, the C-arm fluoroscopy is rotated to the left anterior oblique (LAO) view with removal of parallax, aligning the fluoroscopic angles correctly to avoid visual distortion, making the aortic annulus appear as a perfect circle and the delivery catheter free of angular displacement. Root angiography is then performed. The valve is released when the entire stent is positioned below the annulus plane. If the desired depth is not achieved after root angiography, the valve is recaptured and redeployed at a higher or lower position. All angles and implantation depths below the annulus plane at different projections are recorded and compared.

### Statistics

Univariate analyses were conducted to compare demographic, procedural, and outcome parameters of patients undergoing SEV TAVR using different techniques. Continuous variables are presented as mean ± SD and were compared using either the Student's *t*-test or the Wilcoxon rank sum test. Categorical variables are expressed as counts and percentages and were compared using Pearson's chi-square test or Fisher's exact test, as appropriate. Clinical characteristics and laboratory measurements between the groups were compared using relevant statistical tests.

In multivariate analyses, independent predictors of conduction disturbances (both new LBBB and PPI) and PPI were identified, including device types and variables associated with conduction disturbances or PPI in univariate analyses. The Receiver Operating Characteristic (ROC) curve was used to assess the predictive performance of demographic and procedural parameters for PPI risk. The optimal cutoff point on the ROC curve was determined using Youden's index. A two-sided *p*-value of <0.05 was considered statistically significant for all analyses. Statistical calculations were performed using GraphPad Prism 10.0 software.

## Results

### Baseline characteristics, electrocardiographic and computed tomographic measurements of the patients in this study

In this cohort study, 114 patients received either the Medtronic CoreValve Evolut R™ or the Medtronic Evolut PRO™. Seventeen patients (6 in the COL group and 11 in the CON group) were excluded from the study due to pre-existing pacemakers. Forty-seven patients received SEVs utilizing the COL technique (the COL group), and 50 patients received TAVR using the conventional three-cusp view technique (the CON group). The pre-operative, peri-operative, and post-operative patient demographics, electrocardiographic and CT measurements are shown in Table 1. Overall, the two groups were well matched, with no significant differences observed in baseline characteristics including demographic data and baseline electrocardiographic and CT measurements. However, there were more patients presented with heart failure and the measured left ventricular outflow tract (LVOT) area was significantly larger in the CON group. The length of the membranous septum, measured by either the coronal method or the MIDAS method, also showed no significant difference between the two groups (Table 1).

**Table 1:** Baseline characteristics, electrocardiographic and computed tomographic measurements of the patients in this study.

	CON group (*N* = 50)	COL group (*N* = 47)	*P*-value
Age, years	83 ± 6.5	81 ± 8.6	0.248
Height, cm	155 ± 6.7	156 ± 11	0.442
Weight, kg	60 ± 12	60 ± 11	0.787
Male gender, %	48	40.4	0.541
Heart failure, NYHA Fc III/IV, %	60	34	0.0146
Type 2 diabetes mellitus, %	36	31.9	0.830
Atrial fibrillation, %	22.5	14.9	0.436
Severe leaflet calcification, %	32	36.2	0.675
Electrocardiograms			
First degree AV block, %	22.5	8.51	0.091
Pre-existing LBBB, %	2	0	0.999
Pre-existing RBBB, %	14.3	4.3	0.160
P axis, degrees	52 ± 63	50 ± 36	0.896
R axis, degrees	24 ± 44	24 ± 33	0.952
PR interval, ms	194 ± 47	175 ± 42	0.056
QRS interval, ms	102 ± 20	97 ± 15	0.170
QTc, ms	442 ± 29	451 ± 32	0.157
Multi-slice computed tomography			
Annular perimeter, mm	73 ± 6.3	71 ± 6.4	0.206
Annular area, mm^2^	414 ± 71	389 ± 87	0.122
LVOT perimeter, mm	73 ± 7.1	71 ± 6.7	0.269
LVOT area, mm^2^	405 ± 81	371 ± 87	0.049
Sinus of Valsalva diameter, mm	31 ± 3.2	31 ± 3.2	0.916
STJ diameter, mm	27 ± 3.8	26 ± 3.2	0.447
Ascending aorta diameter, mm	32 ± 3.5	32 ± 3.1	0.315
Aortic root angle, degrees	49 ± 6.5	50 ± 8.5	0.387
LCA height, mm	12 ± 2.7	12 ± 1.9	0.616
RCA height, mm	15 ± 2.4	16 ± 4.2	0.443
Membranous septum length (coronal method), mm	4.6 ± 1.0	4.0 ± 1.9	0.119
Membranous septum length (MIDAS method), mm	2.4 ± 2.0	2.1 ± 1.7	0.415

AV block, atrio-ventricular block; CON, conventional three-cusp; COL, cusp overlap; LBBB, left bundle branch block; LCA, left coronary artery; LVOT, left ventricular outflow tract; MIDAS, minimizing depth according to the membranous septum; NYHA Fc, New York Heart Association functional class; QTc, corrected QT interval; RBBB, right bundle branch block; RCA, right coronary artery; STJ, sino-tubular junction.

### Procedural characteristics

The technical details of the procedures and their outcomes are outlined in Table 2. TAVR procedures were predominantly conducted via transfemoral access. Transcarotid alternative access was used for SEV implantation in 4% of patients in the CON group and 10.6% of patients in the COL group. As mentioned, the Medtronic CoreValve Evolut R™ was more commonly used in the CON group, while the Medtronic Evolut PRO™ was more commonly used in the COL group. Additionally, the requirement for pre- and post-dilatation, and recapture/repositioning was similar in both groups (Table 2).

**Table 2:** Procedural characteristics, and procedural and clinical outcomes of the patients in this study.

	CON group (*N* = 50)	COL group (*N* = 47)	*P*-value
Alternative access, %	4.0	10.6	0.207
Medtronic Evolut PRO™ valve, %	8	91.5	0.0001
Valve size (34 mm), %	4	4.3	0.950
SEV deployment method			
Top-down from annular plane, %	50	4.4	0.0001
Top-down from supra-annular plane, %	4	82.6	0.0001
Bottom-up from LVOT, %	46	13	0.0004
Balloon pre-dilation, %	22	19.1	0.729
Balloon post-dilation, %	46	46.8	0.936
Re-capture/reposition, %	32	40.4	0.388
Implantation depth below NCC			
PRD (LAO view), mm	4.3 ± 1.8	3.4 ± 1.5	0.007
RD (LAO view), mm	3.9 ± 2.6	2.7 ± 2.0	0.021
RD (COL view), mm	–	3.0 ± 1.6	–
Implantation depth below LCC			
PRD (LAO view), mm	7.3 ± 2.8	7.2 ± 2.8	0.904
RD (LAO view), mm	5.7 ± 2.3	4.6 ± 2.2	0.018
RD (COL view), mm	–	4.7 ± 2.3	–
MS length—implantation depth below NCC			
PRD (Coronal method), mm	−0.4 ± 2.6	1.1 ± 2.6	0.006
PRD (MIDAS method), mm	−2.3 ± 2.6	−1.0 ± 2.5	0.017
RD (Coronal method, LAO view), mm	0.1 ± 3.2	1.5 ± 2.8	0.002
RD (MIDAS method, LAO view), mm	−1.8 ± 2.9	−0.5 ± 2.6	0.046
RD (Coronal method, COL view), mm	–	1.6 ± 2.4	–
RD (MIDAS method, COL view), mm	–	−0.5 ± 2.4	–
≧moderate PVL after TAVR, %	6.1	0	0.0881
Conduction disturbances after TAVR			
New LBBB (immediately after TAVR), %	46	27.7	0.062
New LBBB (1 week after TAVR), %	26	8.7	0.027
Recovery from LBBB, %	56.5	69.2	0.501
PPI rate, %	18	4.26	0.033

CON, conventional three-cusp; COL, cusp overlap; LAO, left anterior oblique; LBBB, left bundle branch block; LCC, left coronary cusp; LVOT, left ventricular outflow tract; MIDAS, minimizing depth according to the membranous septum; NCC, non-coronary cusp; PPI, permanent pacemaker implantation; PVL, paravalvular leakage; SEV, self-expanding valve; TAVR, transcatheter aortic valve replacement; PRD, pre-releasing depth; RD: released depth.

In the CON group, 54% of the SEVs were successfully deployed using a top-down approach from either the annular (50%) or supra-annular plane (4%). However, in 46% of patients, the SEVs were successfully deployed using a bottom-up approach, pulling back from the LVOT for better positioning. In contrast, 87% of the SEVs in the COL group were successfully deployed using a top-down approach from either the annular (4.4%) or supra-annular plane (82.6%). Only 13% of patients needed a bottom-up approach for positioning. The differences in SEV deployment approaches were significantly different between the two groups.

### Higher implantation could be achieved with the use of the COL technique compared to the CON technique

**Figure 1:**
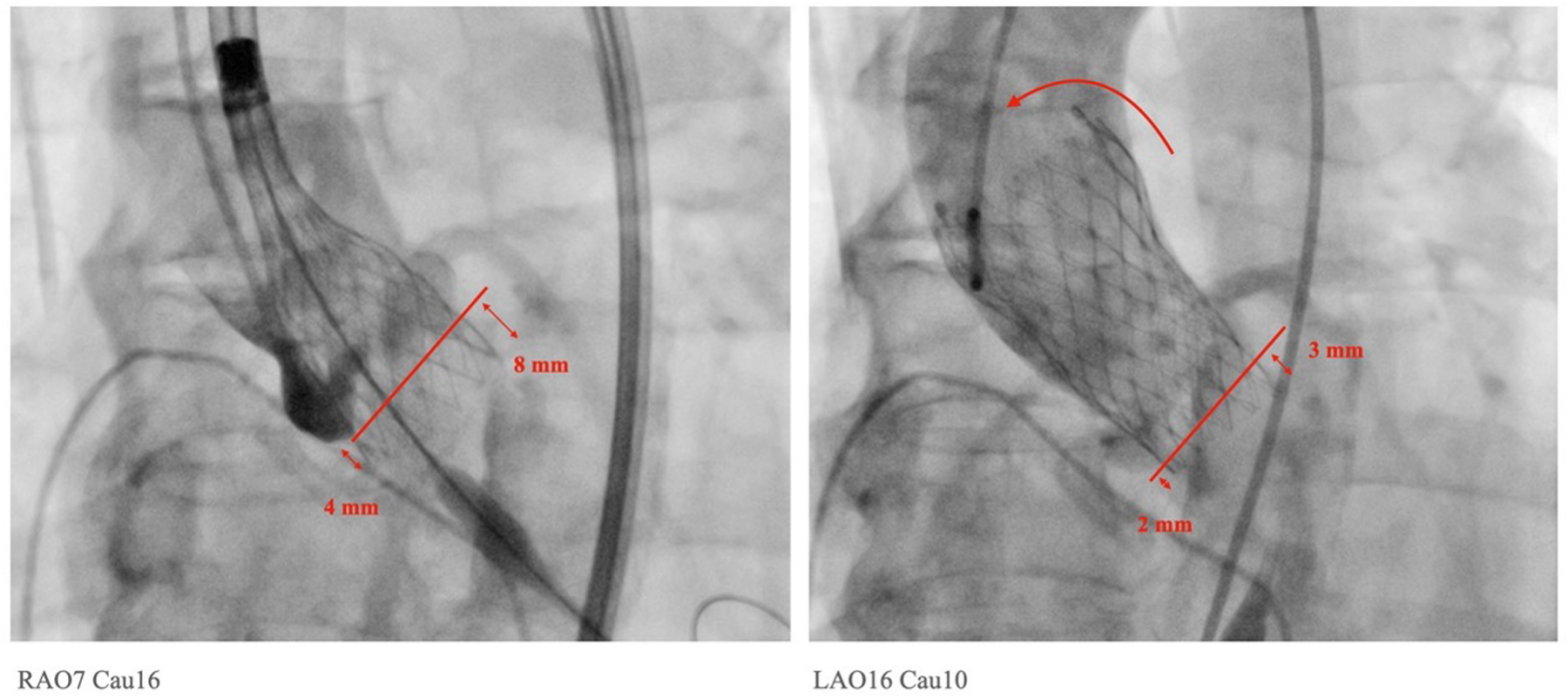
Measurements of implantation depth were taken in the left anterior oblique view with the removal of parallax. The implantation depths before and after final release were measured accordingly. In this particular case, a tilt of the Medtronic Evolut PRO™ with upward displacement was observed, changing from 4 mm to 2 mm on the NCC side and from 8 mm to 3 mm on the LCC side after the final release.

The implantation depth below the NCC is considered to be associated with the PPI rate and the rate of newly developed conduction disturbances after TAVR. We examined and compared the implantation depths below the NCC and LCC before and after the final release of the SEV using the LAO view with the removal of the parallax and the COL view (Table 2; Figure 1). Our findings showed that the implantation depth below the NCC did not change significantly before and after the release of the SEVs (PRD vs. RD = 4.3 ± 1.8 mm vs. 3.9 ± 2.6 mm, *p* = NS in the CON group; PRD vs. RD = 3.4 ± 1.5 mm vs. 2.7 ± 2.0 mm, *p* = NS in the COL group). However, the COL technique resulted in a significant reduction in implantation depth below the NCC both before and after the final release (PRD: CON group vs. COL group = 4.3 ± 1.8 mm vs. 3.4 ± 1.5 mm, *p* = 0.007; RD: CON group vs. COL group = 3.9 ± 2.6 mm vs. 2.7 ± 2.0 mm, *p* = 0.021) (Table 2).

In contrast to the findings on the NCC side, the implantation depth below the LCC changed significantly after release. In the CON group, the depth decreased from 7.3 ± 2.8 mm in the PRD to 5.7 ± 2.3 mm after release (*p* < 0.0001). In the COL group, the depth decreased from 7.2 ± 2.8 mm in PRD to 4.7 ± 2.3 mm in the COL view, and to 4.6 ± 2.2 mm in the LAO parallax view (*p* < 0.0001). Additionally, the COL technique resulted in a significant reduction in implantation depth below the LCC after the final release (PRD: CON group vs. COL group = 7.3 ± 2.8 mm vs. 7.2 ± 2.8 mm, p = NS; RD: CON group vs. COL group = 5.7 ± 2.3 mm vs. 4.6 ± 2.2 mm, *p* = 0.018). These findings indicate that the SEV moved upwards significantly after release on the LCC side, while on the NCC side, there was a slight but insignificant upward displacement after the final release of the SEV. Moreover, the COL technique significantly decreases the implantation depths on both the NCC and LCC sides.

Comparisons between the membranous septum length and the implantation depth below NCC for the two deployment techniques revealed statistical significance. The pre-releasing membranous septum length minus the implantation depth below NCC was significantly shorter in the CON group compared to the COL group, regardless of whether the membranous septum length was measured using the coronal method or the MIDAS method (PRD: CON vs. COL = −0.4 ± 2.6 mm vs. 1.1 ± 2.6 mm, *p* = 0.006; RD: CON vs. COL = 0.1 ± 0.32 mm vs. 1.5 ± 2.8 mm, *p* = 0.002, by coronal method, and PRD: CON vs. COL = −2.3 ± 2.6 mm vs. −1.0 ± 2.5 mm, *p* = 0.017; RD: CON vs. COL = −1.8 ± 2.9 mm vs. −0.5 ± 2.6 mm, *p* = 0.046, by MIDAS method, respectively).

### Significant reduction in LBBB and PPI rates in the COL group following TAVR

**Figure 2:**
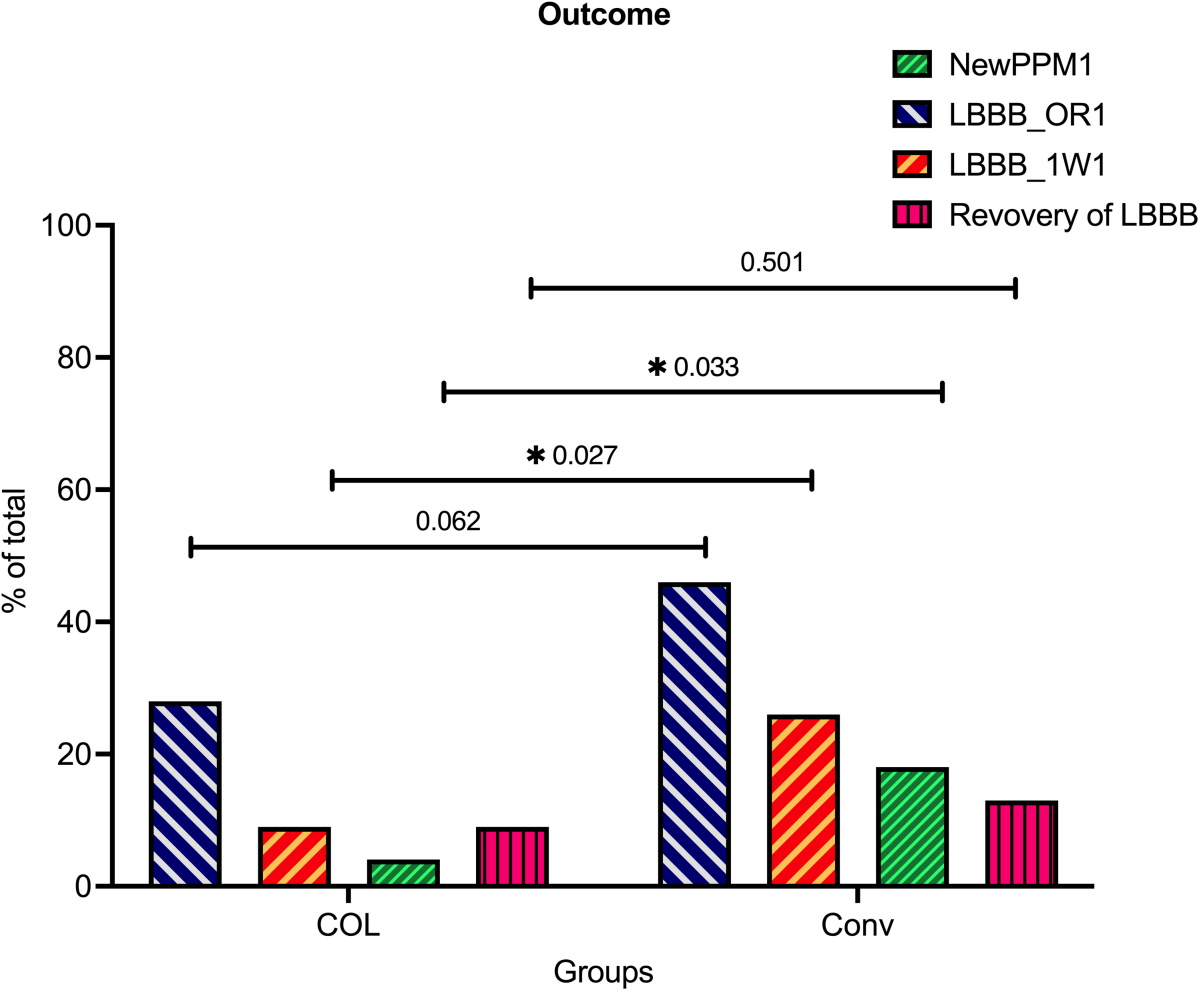
Significantly reduced conduction disturbance rates in the COL group compared to the CON group after TAVR. The incidences of new permanent pacemaker (PPM) implantation, newly developed LBBB in the operating room (new LBBB@OR), newly developed LBBB 1 week after transcatheter aortic valve replacement (TAVR) (new LBBB@1W), and recovery of LBBB 1 week after TAVR were illustrated and compared between the conventional (CON) group and cusp-overlap (COL) group.

Patients in the CON group tended to have a higher incidence of moderate or greater paravalvular leakage after TAVR, although this difference did not reach statistical significance (CON group vs. COL group = 6.01% vs. 0%, *p* = 0.0881) Figure 2; Table 2; Supplementary Video 1.

In terms of conduction disturbances, patients in the COL group demonstrated significantly lower rates of newly developed LBBB both in the operating room and 1 week post-TAVR (immediate LBBB after TAVR: CON vs. COL = 46% vs. 27.7%, *p* = 0.0062; and LBBB 1 week after TAVR: CON vs. COL = 26% vs. 8.7%, *p* = 0.027, respectively). Moreover, the PPI rates 1 week after TAVR were also significantly lower in the COL group compared to the CON group (4.3% vs. 18%, *p* = 0.0329). A proportion of patients who initially presented with LBBB in the operating room showed recovery after 1 week (43.5% in the CON group vs. 61.5% in the COL group, *p* = 0.137).

### Predictors of newly developed LBBB and PPI in as patients undergoing SEV TAVR using the COL technique

Predictors for newly developed LBBB and PPI were further analyzed using univariate and multivariate logistic regression. Independent predictors of newly developed LBBB, PPI, and both were identified using multivariate analyses. Variables with a *p*-value < 0.1 in the univariate analysis were included in the multivariate model (Table 3).

**Table 3:** Predictors of conduction disturbances and permanent pacemaker implantation in aortic stenosis patients undergoing self-expanding valve transcatheter aortic valve replacement using the cusp overlap technique.

	*P*-value (univariate)	*P*-value (multivariate)	Odd ratio	95% C.I.
Predictors of newly developed LBBB and PPI 1 week after TAVR				
PRD below NCC	0.002	0.029	1.588	1.049–2.403
PRD below LCC	0.241			
RD implantation depth below NCC	0.010	0.998	0.999	0.670–1.491
RD rrrrbelow LCC	0.003	0.197	1.348	0.857–2.120
Predictors of PPI 1 week after TAVR				
LVOT perimeter	0.026	0.018	0.867	0.770–0.975
Membranous septum length measured by coronal method	0.039	0.042	0.545	0.303–0.979
PRD below LCC	0.023	0.009	1.490	1.103–2.012

LBBB, left bundle branch block; LCC, left coronary cusp; LVOT, left ventricular outflow tract; NCC, non-coronary cusp; PPI, permanent pacemaker implantation; TAVR, transcatheter aortic valve replacement; PRD, pre-releasing depth; RD, released depth.

Significantly, deeper pre-release and release implantation depths beneath the NCC and LCC were correlated with an increased risk of conduction disturbances. Multivariate analysis further identified the LVOT perimeter, membranous septum length measured via the coronal method, and pre-release implantation depth below the LCC as significant predictors of PPI. The receiver operating characteristic (ROC) curve, along with Youden's index, were utilized to establish optimal thresholds for predicting PPI following transcatheter aortic valve replacement (TAVR). It was determined that a pre-releasing LCC implantation depth greater than 6 mm (ROC area: 0.799, *p* = 0.0028, maximal Youden's index at depth >6.5 mm), a membranous septum length less than 5 mm measured by the coronal method (ROC area: 0.690, *p* = 0.0412, maximal Youden's index at length <4.85 mm), and a difference between coronally measured membranous septum length and implantation depth below the NCC of −1 mm (ROC area: 0.726, *p* = 0.016, maximal Youden's index at length ≤0.9 mm) were the optimal cutoff values for predicting PPI post-TAVR.

## Discussion

The main findings of our study are as follows: (1) higher implantation and less post-TAVR paravalvular leakage were achieved with the use of the COL technique and Evolut PRO™ valve compared to the CON technique and Evolut R™; (2) the pre-releasing and released membranous septum length minus the implantation depth below the NCC was significantly shorter in the CON group compared to the COL group; (3) in terms of conduction disturbances, patients in the COL group demonstrated significantly lower rates of newly developed LBBB and PPI compared to the CON group; (4) multivariate analysis identified LVOT perimeter, membranous septum length measured by the coronal method, and pre-releasing implantation depth below the LCC as significant predictors of PPI after TAVR.

Conduction system abnormalities are the most common complication following TAVR and are associated with increased morbidity and mortality, longer hospital stays, and higher costs of care (12). Key risk factors for conduction disturbances include pre-existing RBBB, the use of certain SEVs, and the depth of device implantation within the LVOT (1–4). In this cohort, pre-existing RBBB was present in 14.3% of the conventional group compared to 4.3% in the cusp overlap group (*p* = 0.167). Univariate and multivariate analyses did not identify pre-existing RBBB as a contributing factor, suggesting that implantation technique may have a greater impact than pre-existing conduction block (13). Strategies to minimize conduction system injury focus on reducing interaction between the transcatheter heart valve and the membranous and muscular septum of the LVOT, where conduction fibers are most superficial. The membranous septum, located at the junction of the NCC and RCC, varies significantly among individuals and can influence the risk of conduction disturbances. Recent outcomes after SEV TAVR have improved significantly with the adoption of the COL technique, which targets a 3 mm inflow implantation depth to optimize valve placement and reduce PPI rates (2–4, 14–16). In this study, we aimed to identify risk factors for patients undergoing SEV TAVR using the COL technique. Our findings demonstrate that higher implantation depths and reduced post-TAVR paravalvular leakage were achieved with the COL technique using the Evolut PRO™ valve compared to the CON technique with the Evolut R™ valve. Additionally, the COL group exhibited significantly lower rates of newly developed LBBB and PPI compared to the CON group. Multivariate analysis identified not only the well-recognized risk factors of final implantation depth and smaller LVOT diameter but also a membranous septum length of less than 5 mm (measured by the coronal method) and a pre-releasing implantation depth below the LCC greater than 6 mm as significant predictors of PPI after TAVR. The depth of implantation relative to the membranous septum length is a critical factor influencing the risk of PPI. Jilaihawi et al. (9) reported that deploying a transcatheter heart valve below the membranous septum increases PPI risk, with the risk rising significantly when the difference between membranous septum length and implantation depth is negative (−1.6 mm in patients requiring pacemakers vs. 0.6 mm in those who did not; *p* < 0.001). Consistent with previous studies, we observed that the membranous septum length minus the implantation depths below the NCC and LCC was significantly shorter in the CON group compared to the COL group, regardless of the measurement method (coronal or MIDAS) (17). Furthermore, we identified a membranous septum length of less than 5 mm and a difference between the coronally measured membranous septum length and the implantation depth below the NCC of less than −1 mm as significant predictors of PPI. Therefore, maintaining SEV implantation depth within 1 mm of the membranous septum, in addition to using the COL technique and targeting a depth of less than 3 mm below the NCC, may significantly reduce the risk of PPI following SEV TAVR.

As mentioned earlier, if the SEV is deployed below the membranous septum, there is a greater chance of interaction with the conduction system (17). Therefore, in contrast to the CON technique, we emphasize a top-down approach for deployment of the SEV, starting with the catheter marker band positioned at the mid-portion or lower third of the pigtail in the NCC. Once the capsule is retracted, the inflow of the nitinol frame advances across the annulus and is positioned 3 mm below. This maneuver avoids traumatic advancement of the SEV into the ventricle, which would necessitate subsequent maneuvers to retract the catheter and increase interaction of the flared end of the SEV and/or the nose cone of the delivery system with the membranous septum. This top-down deployment approach was successfully accomplished in 87% of patients using COL techniques in our series and is considered a critical aspect of SEV deployment.

It is noteworthy that, in addition to the conventionally recognized released implantation depth below the NCC and LCC, we identified that pre-releasing implantation depths below the NCC and LCC were also predictive of newly developed LBBB and PPI. This is particularly true for a pre-releasing LCC implantation depth greater than 6 mm, which can predict PPI risk after TAVR. By using the COL technique, valve displacement on the LCC side is minimized (Table 2). After valve release, LCC depth is no longer a determining factor for conduction system injury, as the correction effect of the COL technique during SEV TAVR minimizes this asymmetry. This occurs because, during deployment of the CoreValve Evolut system, the valve frame is often asymmetrical, with the LCC side typically deeper. This occurs because the CoreValve Evolut delivery system has two spines, causing the valve to tilt towards the NCC side and unfold in a fan shape towards the LCC side when it begins to release. Since the membranous septum may extend partially to the vicinity of the LCC and the His bundle pierces the membranous portion between the NCC and LCC, sometimes following a more posterior path (15), a deeply implanted stent frame on the LCC side can still compress the conduction tissue, leading to conduction disturbances. When the implantation depth on the NCC side is successfully reduced to an average of about 3 mm using the COL technique, the implantation depth of the valve on the LCC side becomes even more critical. It is recommended that, at 80% deployment, implanters should rotate the gantry to a LAO projection to visualize the LCC and ensure that the inflow is neither supra-annular nor more than 6 mm in depth. Once final positioning is confirmed, implanters may retract the left ventricular wire, centralize the nose cone, and slowly release the delivery catheter from the SEV by releasing the frame paddles. The newer iteration of the Medtronic Evolut system (Evolut FX™) may reduce device pivoting upon release and achieve more symmetric implantation depths on both the NCC and LCC when utilizing the COL technique (7).

### Study limitations

The study has several limitations that should be acknowledged. Firstly, the relatively small sample size in both groups and the single-center nature of the study limit the generalizability of the findings. Consequently, caution should be exercised when interpreting the results, especially regarding comparisons between the two techniques. Secondly, this is a before-and-after observational study with inherent limitations, primarily selection and confounding biases. Additionally, the evolution of the heart team's experience and technical refinements over time may have contributed to improved outcomes. However, we have tried to mitigate these biases by recruiting consecutive patients undergoing SEV TAVR during a relatively short period of 3 years.

Therefore, we consider this study useful for assessing the effects of implantation technique changes over time. Furthermore, this study was focused on a particular kind of SEV (Medtronic Evolut system). Therefore, caution should be taken in extrapolating these results. The Medtronic Evolut R™ and Evolut Pro™ systems featured a sealing skirt and delivery sheath diameter. The delivery system, which incorporated two metal spines, demonstrated similar trackability and radial force as the self-expanding valve. According to our observations, these differences did not significantly affect the outcomes. However, the Evolut R™ and Pro™ systems were not widely available in most centers globally. The newer Evolut FX™ system, with a single metal spine, has been shown to have equivalent effectiveness when using the COL technique (17, 18). The use of other self-expanding systems, such as the Abbott Portico system, has yielded similar results. However, modifications were necessary due to changes in the radial force of the valve and the trackability of the delivery system (19). Future studies addressing these limitations, such as larger multicenter randomized controlled trials conducted over a shorter and more homogeneous time frame, are warranted to provide more definitive conclusions regarding the use of the COL technique in SEV TAVR.

## Conclusion

The COL implantation technique is a safe and feasible modification to the SEV TAVR procedure (20). This study showed that using the COL technique significantly reduced the incidence of newly developed LBBB and PPI in SEV TAVR compared to the CON technique. Furthermore, our study recommends maintaining an implantation depth of less than 6 mm below the left coronary cusp (LCC) prior to final release to enhance the controlled ostial landing (COL) technique (Central Illustration). This approach may further reduce the risk of permanent pacemaker implantation (PPI).

**Central Illustration:**
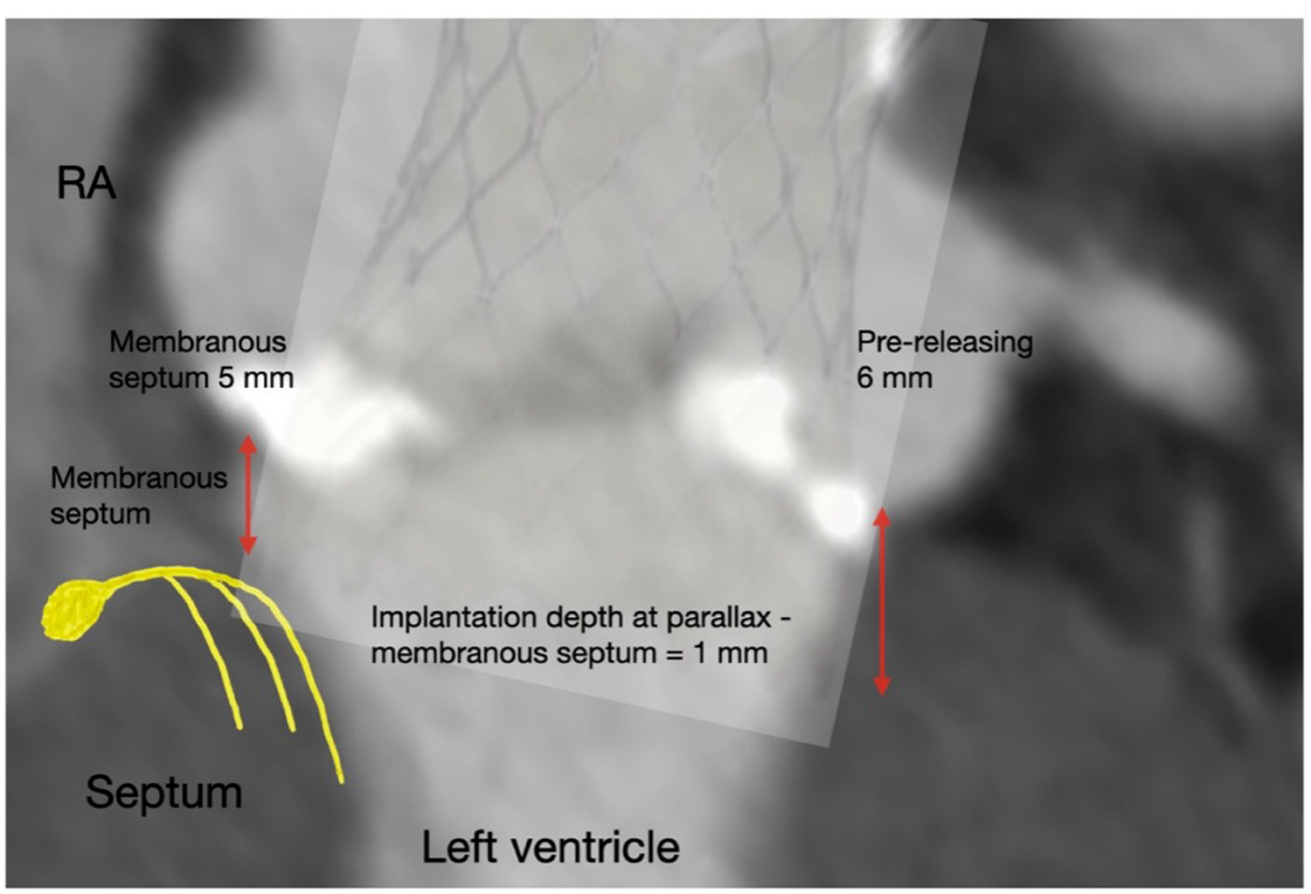
Using the cusp-overlap technique significantly reduces the risk of newly developed LBBB and permanent pacemaker implantation (PPI) after transcatheter aortic valve replacement (TAVR). Refining the technique by keeping the self-expanding valve implantation depth within 1 mm of the membranous septum and maintaining an implantation depth of less than 6 mm below the LCC before final release may further minimize the risk of PPI.

## Data Availability

The datasets presented in this article are not readily available because the data has limited access by IRB. Requests to access the datasets should be directed to youngt_lee@yahoo.com.tw.
